# Safety and Efficacy of Early Rehabilitation With Assistance From a Single-Joint Hybrid Assistive Limb in Patients With Total Knee Arthroplasty: A Randomized Controlled Clinical Pilot Study

**DOI:** 10.7759/cureus.57738

**Published:** 2024-04-06

**Authors:** Takaya Watabe, Muramatsu Ryota, Takuya Sengoku, Yushin Mizuno, Goro Sakurai, Shinya Yoshida, Kentaro Igarashi

**Affiliations:** 1 Rehabilitation, Kanazawa University Hospital, Kanazawa, JPN; 2 Orthopedic Surgery, Kanazawa University, Kanazawa, JPN

**Keywords:** knee range of motion (rom), total knee arthroplasty technique, single-joint hybrid assistive limb, musculoskeletal rehabilitation, knee osteoarthritis

## Abstract

Background

This study aimed to evaluate the safety and effectiveness of knee exercise within four hours after total knee arthroplasty (TKA) using a single-joint hybrid assistive limb (HAL-SJ).

Materials and methods

This pilot single-blind randomized controlled trial included participants who underwent TKA for osteoarthritis and were randomly allocated to the early rehabilitation (n = 14) or control rehabilitation (n = 16) group. Knee rehabilitation exercises using the HAL-SJ began within four hours postoperatively in the early group and seven days after surgery in the control group. Knee circumference, range of motion (ROM), pain, muscle strength, and extension lag were assessed before and one and two weeks after surgery.

Results

Circumferences at 1 and 10 cm from the upper edge of the patella did not differ between the groups before surgery or one week postoperatively. The extension lag and knee flexion ROM after one week were significantly better in the early intervention group than in the control group. However, the quadriceps and hamstring isometric knee strength and pain scores did not differ between the groups at one and two weeks postoperatively. HAL-SJ-related complications were not reported.

Conclusion

Rehabilitation knee exercises using the HAL-SJ within four hours after TKA improved extension lag and knee flexion ROM without exacerbating knee swelling and pain.

## Introduction

Total knee arthroplasty (TKA) is one of the most common surgical procedures for treating knee osteoarthritis (OA). The incidence of OA, and thus TKA, is increasing worldwide, increasing the burden on healthcare systems [1]. Early rehabilitation within 24 hours of TKA is associated with a shorter hospital stay, lower overall cost, reduced pain, improved range of motion (ROM), and improved muscle strength [2,3]. Additionally, Kubota et al. [4] demonstrated that early rehabilitation within four hours of TKA reduced early extension ROM loss and pain and improved the gait pattern compared with rehabilitation within two days of TKA. Therefore, early rehabilitation is crucial for improving knee joint function after TKA.

In recent years, the single-joint hybrid assistive limb (HAL-SJ; Cyberdyne Inc., Tsukuba, Japan) [5] has attracted attention as a novel rehabilitation device for use after TKA [6]. The HAL-SJ is a neurologically controlled therapy device supporting knee joint flexion and extension triggered by a patient’s bioelectrical signals (BES) [7,8]. Previous studies have conducted knee joint extension training using the HAL-SJ after TKA and reported improved extension lag and gait function without increasing knee pain [6,9,10].

Early rehabilitation may be associated with safety concerns such as pain, increased knee swelling, and wound dehiscence. However, the safety and efficacy of early rehabilitation using HAL-SJ assistance in patients who have undergone TKA remain unclear. Therefore, this study evaluated the safety and efficacy of knee exercise within four hours after TKA using the HAL-SJ. We hypothesized that early initiation of knee exercises using the HAL-SJ would enhance knee joint function and reduce pain.

## Materials and methods

Study design

This was a pilot single-blind randomized controlled trial (RCT). The trial has been reported according to the Consolidated Standards of Reporting Trials (CONSORT) 2010 statement: extension for pilot/feasibility studies [11]. The participants were blinded to the group allocation to ensure that the allocation did not influence adherence to the protocol or increase dropout risk. A randomized sequence was generated using Microsoft Excel for Mac (version 16.82; Microsoft Corporation, Redmond, Washington, DC). The Ethics Review Committee of our institution approved this study (113786), which was registered with the University Hospital Medical Information Network on February 21, 2024 (UMIN000053675). The physiotherapists could not be blinded to the allocation but were encouraged to deliver both interventions with equal enthusiasm.

Patient recruitment

**Figure 1:**
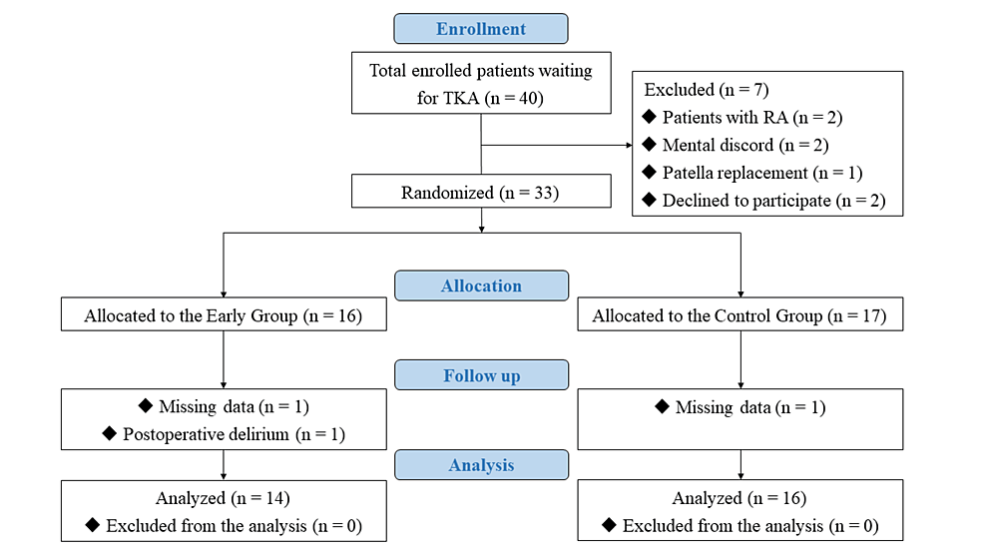
Patient selection flow diagram. RA, Rheumatoid arthritis; TKA, Total knee arthroplasty

Between June 2021 and August 2022, 40 patients with knee OA underwent primary TKA at our institution using the Robotic Surgical Assistant Knee System (Zimmer Biomet, Warsaw, IN), and an under-vastus approach was performed by two surgeons (Figure 1). The strict exclusion criteria were as follows: rheumatoid arthritis, severe cognitive deficits or mental disorders, a Kellgren-Lawrence score of <2, lower extremity paralysis, previous tibial osteotomy, revision arthroplasty, body mass index >35 kg/m^2^, pain that makes conventional rehabilitation impossible, non-device-related revision surgery, and refusal to participate by the patient. Seven patients who met one or more of these criteria were excluded (Table 1); 33 patients were included and assigned to the early rehabilitation (HAL-SJ exercises within four hours of TKA) or control (HAL-SJ exercises seven days after TKA) groups. 

**Table 1:** Patient demographics data (mean ± SD). K/L grade, Kellgren–Lawrence grade; SD, Standard deviation

Characteristics	Early group	Control group	p-value
n	14	16	―
Males: Females	5:9	3:13	0.263
Age (years)	75.6 ± 5.6	75.1 ± 6.1	0.834
Height (cm)	155.7 ± 10.8	156.1 ± 7.1	0.923
Weight (kg)	62.1 ± 9.1	62.5 ± 8.7	0.903
K/L grade	Ⅳ:12, Ⅲ:2	Ⅳ13, Ⅲ:3	0.567

Measurements

The primary outcome parameters were assessed preoperatively and one and two weeks postoperatively, including the knee circumference 1 and 10 cm from the upper edge of the patella, quadricep and hamstring isometric knee strength, the passive ROM of the affected knee joint (flexion and extension), and the numeric rating scale (NRS) pain score; licensed physical therapists evaluated the primary outcomes. The secondary outcome parameters included HAL-SJ-related complications, such as effects on wound healing, soft tissue injuries (e.g., muscle fiber tears), and severe complications (e.g., periprosthetic fractures or implant loosening); orthopedic surgeons assessed the secondary outcomes.

Knee circumference was measured with the participants relaxed in the supine position with the knees extended. Measurements were taken 1 and 10 cm proximal to the upper edge of the patella using a non-stretchable tape measure. Two measurements were performed, and the mean of the two recordings was used for analysis. Previous studies have demonstrated excellent intra-rater and good inter-rater reliabilities for circumference measurements using a tape measure [12]. Quadriceps and hamstring strengths were measured using a handheld dynamometer (HHD; μTas F-1; ANIMA Co., Tokyo, Japan). HHD assessments were conducted using a technique validated for measuring quadriceps and hamstring strength after TKA [13,14]. Isometric knee force was measured as follows: (1) the patient was seated with 90° hip flexion and 90° knee flexion with the hands on their lap; (2) the physical therapists fixed the dynamometer approximately 5 cm proximal to the medial malleolus; and (3) the patient exerted a maximal isometric knee force, while the physical therapists matched the force of the patient with the dynamometer. The HHD force values were measured in newtons and subsequently normalized as a percentage of the patient’s body weight (newton/body weight). The HHD force was measured twice within each assessment period, and the maximum values were used for the analysis. This assessment technique has excellent test-retest reliability (intraclass correlation = 0.91) for patients undergoing TKA [13]. Passive ROM was measured in 5-degree increments using a goniometer (Toudaisiki Goniometer; OG Wellness Co., Ltd., Okayama, Japan). Knee pain was assessed using the NRS on a scale of 0-10 (0 indicating no pain and 10 indicating the worst pain).

Treatment protocol

**Figure 2:**
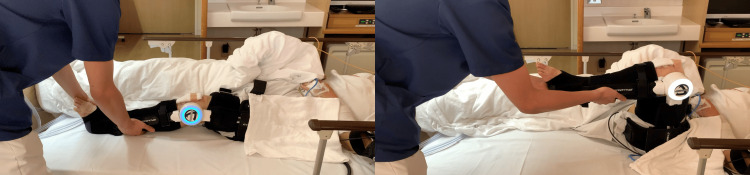
Single-joint hybrid assistive limb training within four hours after surgery. The patient lies in their bed and performs extension and flexion exercises using the affected knee joint.

**Figure 3:**
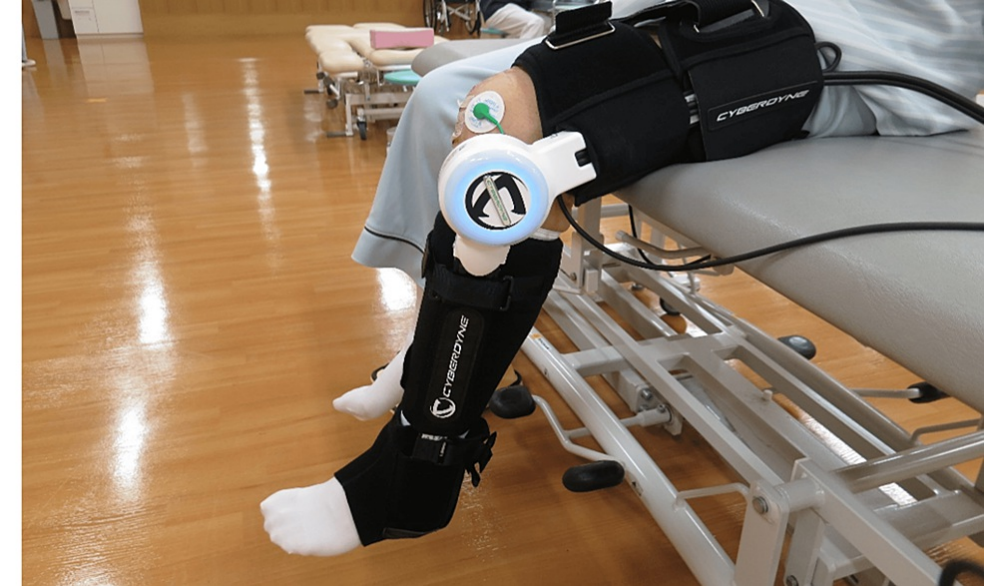
Patient during exercise with single-joint hybrid assistive limb attached to the operated knee from one week after total knee arthroplasty (TKA). Patients in the early assistance and control groups sit in bed and perform extension and flexion exercises with the affected knee joint using the single-joint hybrid assistive limb.

All patients received 40 minutes of rehabilitation per day, five times per week, during their hospitalization. The rehabilitation exercise therapy protocol was the same for all patients; however, the HAL-SJ was started within the first four hours after TKA in the early intervention group and seven days after TKA in the control group. Postoperative analgesic management did not differ between the groups. The anesthesiologist administered an adductor canal block using 20 mL ropivacaine (7.5 mg) during surgery, provided intravenous opioid analgesics as necessary on the first day after surgery, and prescribed oral celecoxib at a dose of 200 mg/day for one week starting the following day. The rehabilitation protocol was as follows: on day 0 (surgery day), patients in the control group remained at rest in bed and received no rehabilitation treatments. In the early intervention group, rehabilitation commenced within four hours after surgery and consisted mainly of active-assisted mobilization using the HAL-SJ. The BES of the vastus medialis and biceps femoris muscles were detected using electrodes that were placed based on the Surface Electromyography for Non-invasive Assessment of Muscles guidelines [15]. In the cybernic voluntary control mode (AutoEXT/AutoFlx), the patient was supported by BES triggered during the extension and flexion of the knee joint. The settings were as follows: extension/flexion signal balance of flexion at 80% and extension at 100%, assist gain adjusted between 20 and 40%, and a torque limit of 50%. The patient was laid in bed, and extension and flexion movements of the affected knee joint were performed (Figure 2). Knee joint movement was assisted. Flexion initially started at 90° but increased as soon as the patient could perform more repetitions; subsequently, it increased to 120°. Five sets of 10 repetitions were performed once daily (rest between sets: one minute; total duration: approximately 15-20 minutes per unit) [5]. Furthermore, both groups received standard treatments to prevent deep vein thrombosis, including calf muscle air pumping and self-ankle dorsiflexion-plantar flexion exercises. On day one, both groups initiated exercises in a seated position in bed, wheelchair transfers, standing, short-distance walking, flexion-extension exercises, and isotonic muscle contraction while seated. From day three until discharge, the activities included active-resisted quadriceps exercises, progressive walking exercises with aids, increased walking distance, gait reeducation, and daily life adaptation activities. From day seven until discharge, both groups performed knee joint extension and flexion exercises for the affected knee using the HAL-SJ while sitting in bed (Figure 3). Each session comprised five sets of 10 repetitions, performed three times a week, with a one-minute rest between sets, totaling 15-20 minutes per session [5].

Statistical analyses

Statistical analysis was performed by using a Shapiro-Wilk test to assess for data normality. For datasets that were found to be normally distributed, an independent t-test was used for analysis. Non-normally distributed data were analyzed with a Mann-Whitney U test. Categorical data were analyzed using chi-square tests. Cohen’s d was used to calculate the effect size for the statistically significant results. Small, medium, and large effect sizes were indicated by Cohen’s d values greater than 0.2, 0.5, and 0.8, respectively [16]. All statistical analyses were performed using R software (version 4.1.2; The R Foundation for Statistical Computing, Vienna, Austria); statistical significance was set at 0.05.

## Results

The sample size of 33 was not formally determined; instead, it was based on a previous pilot RCT that evaluated health professional-guided interventions for musculoskeletal conditions [17-19], which was deemed sufficient for assessing the feasibility criteria. Participants who completed the baseline and follow-up evaluations were included in the analysis, as recommended by the Consolidated Standards of Reporting Trials (CONSORT) guidelines [11]. However, three patients (two in the early group and one in the control group) dropped out because of missing data (n = 2) and postoperative delirium (n = 1). Therefore, 30 patients (14 in the early group and 16 in the control group) were included in the final evaluation. Patient demographic and general data were comparable in both groups (Table 1).

The circumferences 1 and 10 cm from the upper edge of the patella did not differ between the groups preoperatively or one week after TKA (Table 2). Extension lag (7.0 ± 2.7 vs. 11.0 ± 4.8 days, p = 0.019) and knee flexion ROM (102.8 ± 11.2° vs. 93.7 ± 9.4°, p = 0.022) one week after TKA significantly improved in the early group compared to those in the control group. However, the quadriceps and hamstring isometric knee strength and NRS pain scores did not differ between the groups one and two weeks postoperatively. Furthermore, the hospitalization duration and total days of inpatient rehabilitation (excluding weekends) did not differ between the groups before and after surgery (Table 3). We faced no complications associated with HAL-SJ.

**Table 2:** Knee circumference of groups (mean ± SD). ^a^From the upper edge of the patella. SD, Standard deviation; TKA, Total knee arthroplasty

Circumference^a^	Pre-TKA			
	Early group	Control group	p-value	Cohen’s d
1 cm	39.5 ± 1.8	38.7 ± 2.3	0.917	0.38
10 cm	42.0 ± 1.2	42.1 ± 2.9	0.626	0.04
Circumference^a^	Week 1 after TKA			
	Early group	Control group	p-value	Cohen’s d
1 cm	40.8 ± 2.3	41.8 ± 2.8	0.410	0.39
10 cm	44.7 ± 1.3	45.1 ± 3.6	0.728	0.06

**Table 3:** Postoperative clinical results of groups (mean ± SD). * p <0.05. NRS, Numeric rating scale; ROM, Range of motion; SD, Standard deviation; TKA, Total knee arthroplasty

Clinical results	Early group	Control group	p-value	Cohen’s d
Hospitalization duration (days)	17.5 ± 3.3	18.3 ± 3.1	0.441	0.24
Inpatient rehabilitation (days)	12.1 ± 2.4	11.6 ± 1.7	0.654	0.24
Extension lag (days)	7.0 ± 2.7	11.0 ± 4.8	0.019*	1.02
Pre-TKA				
Flexion ROM (°)	110.0 ± 16.0	115.3 ± 16.5	0.492	0.32
Extension ROM (°)	–7.8 ± 4.6	–11.2 ± 11.0	0.286	0.40
Quadriceps strength (N/kg)	2.8 ± 1.2	2.9 ± 0.8	0.436	0.09
Hamstring strength (N/kg)	1.6 ± 0.4	2.0 ± 0.5	0.451	0.88
Resting pain NRS	3.8 ± 1.2	4.8 ± 1.1	0.140	0.86
Week 1 after TKA				
Flexion ROM (°)	102.8 ± 11.2	93.7 ± 9.4	0.022*	0.88
Extension ROM (°)	–2.8 ± 3.2	–3.7 ± 2.8	0.653	0.30
Resting pain NRS	2.2 ± 1.6	2.6 ± 1.5	0.578	0.25
Walking pain NRS	3.7 ± 1.2	4.2 ± 1.5	0.623	0.37
Week 2 after TKA				
Flexion ROM (°)	115.3 ± 9.7	111.8 ± 8.7	0.647	0.19
Extension ROM (°)	–1.4 ± 3.0	–1.5 ± 3.0	0.215	0.10
Quadriceps strength (N/kg)	2.2 ± 1.1	2.3 ± 0.6	0.458	0.24
Hamstring strength (N/kg)	1.3 ± 0.5	1.4 ± 0.4	0.137	0.22
Resting pain NRS	1.0 ± 1.4	1.5 ± 1.2	0.569	0.38
Walking pain NRS	2.1 ± 1.2	2.6 ± 1.3	0.443	0.40

## Discussion

The results of this study support our hypothesis that early initiation of knee exercises using the HAL-SJ enhances knee joint function. To the best of our knowledge, this is the first report to demonstrate the safety and feasibility of the HAL-SJ for orthopedic rehabilitation in the early postoperative period after TKA.

A previous study reported that the HAL-SJ was difficult to use when a continuous femoral nerve block was performed for postoperative pain management after TKA [5]. Quadricep weakness, which increases the risk of falling, is a known complication of femoral nerve blocks [20]. However, associations between the adductor canal block, which blocks the saphenous nerve, and significantly less knee buckling post-TKA and less quadriceps strength weakness have been reported [21,22]. Therefore, using an adductor canal block in this study might have allowed the use of the HAL-SJ within four hours postoperatively without exacerbating pain. In addition, knee circumference and resting and walking NRS pain scores after analgesics did not differ between the groups, and wound dehiscence and deep vein thrombosis were not observed in either group. Intraarticular blood accumulation and quadriceps muscle swelling immediately after TKA caused knee swelling, as assessed by knee circumference [23]. Knee swelling has been associated with exacerbated knee pain and quadriceps weakness [24,25]. In this study, the HAL-SJ use within four hours postoperatively did not affect knee swelling; thus, it might have also reduced knee pain. Furthermore, most patients in the early rehabilitation group did not complain of pain on the same day as the TKA or the following day, probably because of residual adductor canal block. Thus, using the HAL-SJ for rehabilitation during the early postoperative period after TKA will likely have good safety characteristics without adverse events.

This study’s primary finding is that the HAL-SJ group exhibited a significantly improved early extension lag compared with the control group. Previous studies have reported that HAL-SJ-based training is safe and effective, leading to instantaneous improvement in extension lag without worsening knee joint pain [9,10,26,27]. One of the leading causes of extension lag may be quadriceps femoris dysfunction due to abnormal reflexes [28]. In addition, pain from surgery, damage to soft tissues, and swelling inhibit normal knee joint movements [4,29]. The BES generated when the patient attempted to extend the knee joint was detected by the HAL-SJ, which synchronized the extension with the intended motion. Therefore, using the HAL-SJ within four hours postoperatively significantly improved the extension lag, potentially achieving early neuromuscular facilitation and motor learning. However, because the follow-up period was short, the results should be interpreted with clinical caution.

Moreover, patients in the early intervention group had better knee flexion ROM one week after surgery than those in the control group. An early start to knee joint mobilization after TKA improves knee ROM, according to previous studies [2,4]. Thus, active-assisted mobilization using the HAL-SJ with knee flexion from 90° to 120° within four hours after surgery likely contributed to the early improvements in flexion ROM. Furthermore, most patients in the early rehabilitation group did not complain of pain on the day of surgery or the day after surgery, probably because of residual intraoperative anesthesia. Therefore, early HAL-SJ training can be considered a painless ROM exercise that commences within four hours of surgery. 

Limitations

Our study has some limitations. First, the small sample size might have influenced the results. Our pilot study was conducted over 14 months, and we identified 40 potential participants; however, only 30 (75%) were eligible. A 10° passive ROM difference between the groups was considered sufficiently clinically important to increase the treatment frequency. The sample size required to detect a significant difference (two-sided, α <0.05) of at least 10° ROM between groups, with an assumed standard deviation of 15° and a power of 0.80, was 37 participants per group [4]. Therefore, at least 99 participants were required for a sample size of 74. This information will assist in recruitment planning and timelines for future studies. Second, our follow-up period was short; therefore, future studies with extended follow-up periods are necessary to obtain long-term results. Thus, although this preliminary study presented positive results, an RCT with more patients and a longer follow-up period is necessary. Finally, all patients were recruited from a single university hospital, with some regional selection bias. However, this university hospital admits patients from several regions, which we believe increases the generalizability of our findings beyond the studied population.

## Conclusions

Using the HAL-SJ within four hours of TKA is safe and feasible and does not exacerbate knee swelling or pain. Therefore, utilizing the HAL-SJ for rehabilitation in the early postoperative period after TKA will likely demonstrate safe properties with no adverse events.

## References

[1] Cui A, Li H, Wang D, Zhong J, Chen Y, Lu H (2020). Global, regional prevalence, incidence and risk factors of knee osteoarthritis in population-based studies. EClinicalMedicine.

[2] Labraca NS, Castro-Sánchez AM, Matarán-Peñarrocha GA, Arroyo-Morales M, Sánchez-Joya Mdel M, Moreno-Lorenzo C (2011). Benefits of starting rehabilitation within 24 hours of primary total knee arthroplasty: randomized clinical trial. Clin Rehabil.

[3] Iwakiri K, Ohta Y, Shibata Y, Minoda Y, Kobayashi A, Nakamura H (2020). Initiating range of motion exercises within 24 hours following total knee arthroplasty affects the reduction of postoperative pain: a randomized controlled trial. Asia Pac J Sports Med Arthrosc Rehabil Technol.

[4] Kubota M, Kokubo Y, Miyazaki T (2022). Effects of knee extension exercise starting within 4 h after total knee arthroplasty. Eur J Orthop Surg Traumatol.

[5] Mrotzek SJ, Ahmadi S, von Glinski A, Brinkemper A, Aach M, Schildhauer TA, Cibura C (2022). Rehabilitation during early postoperative period following total knee arthroplasty using single-joint hybrid assistive limb as new therapy device: a randomized, controlled clinical pilot study. Arch Orthop Trauma Surg.

[6] Fukaya T, Mutsuzaki H, Yoshikawa K, Koseki K, Iwai K (2021). Effect of training with the hybrid assistive limb on gait cycle kinematics after total knee arthroplasty. Geriatr Orthop Surg Rehabil.

[7] Aach M, Cruciger O, Sczesny-Kaiser M (2014). Voluntary driven exoskeleton as a new tool for rehabilitation in chronic spinal cord injury: a pilot study. Spine J.

[8] Fisahn C, Aach M, Jansen O (2016). The effectiveness and safety of exoskeletons as assistive and rehabilitation devices in the treatment of neurologic gait disorders in patients with spinal cord injury: a systematic review. Global Spine J.

[9] Goto K, Morishita T, Kamada S (2017). Feasibility of rehabilitation using the single-joint hybrid assistive limb to facilitate early recovery following total knee arthroplasty: a pilot study. Assist Technol.

[10] Yoshikawa K, Mutsuzaki H, Sano A, Koseki K, Fukaya T, Mizukami M, Yamazaki M (2018). Training with hybrid assistive limb for walking function after total knee arthroplasty. J Orthop Surg Res.

[11] Moher D, Hopewell S, Schulz KF (2012). CONSORT 2010 explanation and elaboration: updated guidelines for reporting parallel group randomised trials. Int J Surg.

[12] Meijer RS, Rietman JS, Geertzen JH, Bosmans JC, Dijkstra PU (2004). Validity and intra- and interobserver reliability of an indirect volume measurements in patients with upper extremity lymphedema. Lymphology.

[13] Kittelson AJ, Christensen JC, Loyd BJ, Burrows KL, Iannitto J, Stevens-Lapsley JE (2021). Reliability, responsiveness, and validity of handheld dynamometry for assessing quadriceps strength in total knee arthroplasty. Disabil Rehabil.

[14] Koblbauer IF, Lambrecht Y, van der Hulst ML, Neeter C, Engelbert RH, Poolman RW, Scholtes VA (2011). Reliability of maximal isometric knee strength testing with modified hand-held dynamometry in patients awaiting total knee arthroplasty: useful in research and individual patient settings? A reliability study. BMC Musculoskelet Disord.

[15] Hermens HJ, Freriks B, Cartherine DK, Rau G (2000). Development of recommendations for SEMG sensors and sensor placement procedures. J Electromyograph Kinesiol.

[16] Cohen J (1992). A power primer. Psychol Bull.

[17] Tan JM, Menz HB, Crossley KM (2019). The efficacy of foot orthoses in individuals with patellofemoral osteoarthritis: a randomised feasibility trial. Pilot Feasibility Stud.

[18] Patterson BE, Barton CJ, Culvenor AG, Cooper RL, Crossley KM (2021). Exercise-therapy and education for individuals one year after anterior cruciate ligament reconstruction: a pilot randomised controlled trial. BMC Musculoskelet Disord.

[19] Kemp JL, Coburn SL, Jones DM, Crossley KM (2018). The physiotherapy for femoroacetabular impingement rehabilitation study (physioFIRST): a pilot randomized controlled trial. J Orthop Sports Phys Ther.

[20] Kuang MJ, Xu LY, Ma JX, Wang Y, Zhao J, Lu B, Ma XL (2016). Adductor canal block versus continuous femoral nerve block in primary total knee arthroplasty: a meta-analysis. Int J Surg.

[21] Fujita Y, Mera H, Watanabe T (2022). Significantly earlier ambulation and reduced risk of near-falls with continuous infusion nerve blocks: a retrospective pilot study of adductor canal block compared to femoral nerve block in total knee arthroplasty. BMC Musculoskelet Disord.

[22] Fillingham YA, Hannon CP, Kopp SL (2022). The efficacy and safety of regional nerve blocks in total knee arthroplasty: systematic review and direct meta-analysis. J Arthroplasty.

[23] Kubo Y, Sugiyama S, Takachu R (2020). Association between serum n-3 polyunsaturated fatty acids and quadriceps weakness immediately after total knee arthroplasty. PLoS One.

[24] Jang S, Lee K, Ju JH (2021). Recent updates of diagnosis, pathophysiology, and treatment on osteoarthritis of the knee. Int J Mol Sci.

[25] Kim D, Park G, Kuo LT, Park W (2018). The effects of pain on quadriceps strength, joint proprioception and dynamic balance among women aged 65 to 75 years with knee osteoarthritis. BMC Geriatr.

[26] Yoshioka T, Kubota S, Sugaya H, Arai N, Hyodo K, Kanamori A, Yamazaki M (2021). Feasibility and efficacy of knee extension training using a single-joint hybrid assistive limb, versus conventional rehabilitation during the early postoperative period after total knee arthroplasty. J Rural Med.

[27] Yoshioka T, Sugaya H, Kubota S, Onishi M, Kanamori A, Sankai Y, Yamazaki M (2016). Knee-extension training with a single-joint hybrid assistive limb during the early postoperative period after total knee arthroplasty in a patient with osteoarthritis. Case Rep Orthop.

[28] Rice DA, McNair PJ (2010). Quadriceps arthrogenic muscle inhibition: neural mechanisms and treatment perspectives. Semin Arthritis Rheum.

[29] Kubo Y, Sugiyama S, Takachu R, Sugiura T, Sawada M, Kobori K, Kobori M (2020). Effects of preoperative low-intensity training with slow movement on early quadriceps weakness after total knee arthroplasty in patients with knee osteoarthritis: a retrospective propensity score-matched study. BMC Sports Sci Med Rehabil.

